# The Changing Geometry of a Fitness Landscape Along an Adaptive Walk

**DOI:** 10.1371/journal.pcbi.1003520

**Published:** 2014-05-22

**Authors:** Devin Greene, Kristina Crona

**Affiliations:** University of California, Merced, Merced, California, United States of America; Buck Institute for Research on Aging, Novato, United States of America

## Abstract

It has recently been noted that the relative prevalence of the various kinds of epistasis varies along an adaptive walk. This has been explained as a result of mean regression in NK model fitness landscapes. Here we show that this phenomenon occurs quite generally in fitness landscapes. We propose a simple and general explanation for this phenomenon, confirming the role of mean regression. We provide support for this explanation with simulations, and discuss the empirical relevance of our findings.

## Author Summary

The main result concerns the changing geometry along an adaptive walk in a fitness landscape. An adaptive walk is described by a sequence of genotypes of increasing fitness, where two consecutive genotypes differ by a point mutation. We compare patterns of epistasis, or gene interactions, along adaptive walks. Roughly, epistasis is antagonistic (rather than synergistic) if the double mutant combining two beneficial mutations has lower fitness than expected. In the extreme case that the double mutant has lower fitness than one (or both) of the single mutants, one has sign epistasis. We claim that the further one is along an adaptive walk, the larger the frequency of sign epistasis and the smaller the relative amount of antagonistic epistasis relative to synergistic epistasis. We provide a simple and general argument for our claim, which hence likely applies to empirical fitness landscapes. Our claims can readily be checked by empirical biologists. Potential theoretical progress related to our work includes a better understanding of the role of recombination in evolution.

## Introduction

Darwinian evolution can be illustrated as an uphill or adaptive walk in a multidimensional landscape, where one dimension (height) corresponds to genotype fitness, and the geometry of the remaining dimensions is determined by the locus–wise mutational distances between the genotypes. The metaphor of a fitness landscape was introduced by [1], and has been formalized in various ways, see e.g. [2] for a discussion. The fitness landscapes we consider here are called genotypic. A very basic type of a fitness landscape is one where mutation at a locus has a uniform effect regardless of the state of the other loci (or *background* in the usual parlance). In most models, this effect is either additive or multiplicative. Deviations from this basic type occur when the effect on fitness of a mutation at a particular locus is dependent of the state of the other loci. The general term for such background dependence is *epistasis*. We study how epistasis varies along an adaptive walk in a fitness landscape. The topic is important for understanding how a population adapts after a recent change in the environment. Several empirical studies [3], [4] suggest that the adaptation process changes character over time, and the role of epistasis may be critical. The description of the changing form of epistasis given in [5] is the starting point for this work.

**Figure 1:**
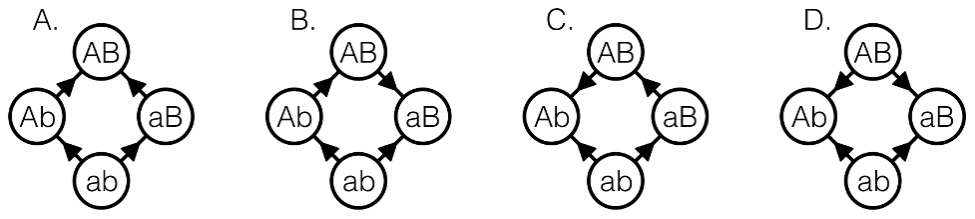
Two biallelic loci corresponds to four genotypes. The fitness relations between neighbors are illustrated in the graphs, where each arrow points toward the genotype with higher fitness. There four possible cases our represented in parts A, B, C and D.

To simplify our discussion, we will restrict ourselves to the following model. A fitness landscape consists of all possible genotypes with a finite number of loci, denoted 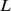, each biallelic, together with the fitnesses of the genotypes. In this manner, we have a one–to–one correspondence between the set of possible genotypes and the set of bit strings of length 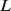. Fitnesses of genotypes are taken to be multiplicative, in the sense that the ratio of fitnesses of one genotype compared to another is the relative reproductive success of the fitter compared to the less fit. In this study, epistasis will be a feature associated with a quadruple of genotypes which differ by at most two loci. When considering such quadruples we will denote one genotype as a base, 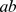, two single mutants 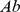 and 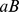, and the double mutant 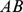. If it is assumed that 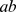 has lowest fitness of the four, we can represent the fitness relations among the four genotypes by the graphs shown in Figure 1.

**Figure 2:**
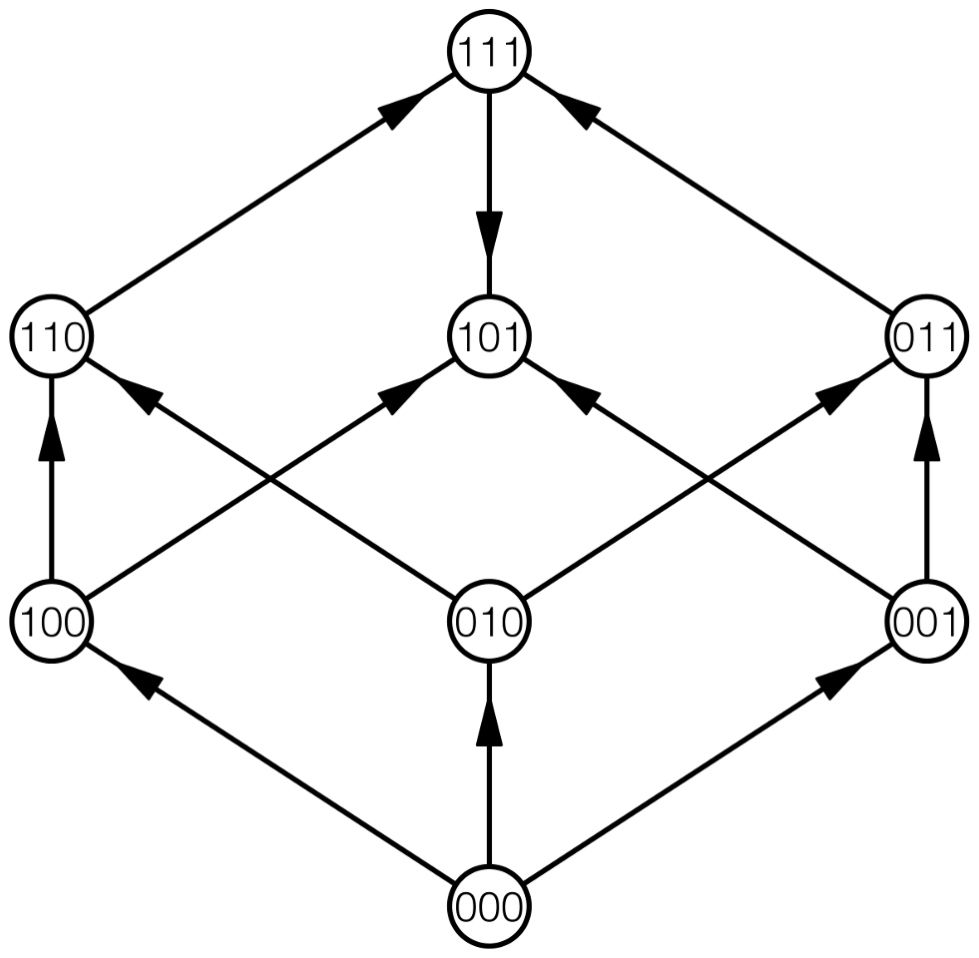
A fitness graph for three loci.

Fitness graphs provided an intuitive way of representing a fitness landscape or its parts. The vertices of the fitness graph represent genotypes. Arrows connect mutational neighbors, with the arrow pointing toward the genotype of higher fitness. Figure 2 shows a fitness graph for 3 loci, and the construction is similar for any number of loci. An adaptive walk can be viewed as a path in the graph respecting the direction of the arrows. Fitness graphs have been used for displaying empirical data [6], [7], and for deriving theoretical results [8], [9].

Cases B, C, and D in Figure 1 present a situation where a mutation at one locus changes the direction of the fitness effect of a mutation at the other locus. Quadruples of genotypes which exhibit one of these relationships are said to exhibit *sign* epistasis, a widely used concept first introduced in [10]. For more background relevant in this context, see e.g. [8], [9], [11], [12]. Several studies of empirical fitness landscapes concern antimicrobial drug resistance, where sign epistasis seems to occur for most landscapes where 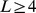 (see e.g. [13] for a survey of empirical fitness landscapes.)

The type of non–sign epistasis in case A of Figure 1 is determined by the sign of the quantity 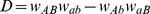, where 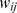 is the fitness of the genotype 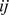. When 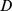 is positive, the quadruple is said to have *synergistic* epistasis, when negative, *antagonistic* epistasis. Conceptually, synergistic epistatis occurs when genotype 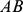 has superior fitness to what would be expected under a multiplicative model based on the fitnesses of 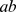, 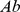, and 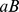, while antagonistic epistasis occurs when 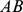 has inferior fitness to what would be expected. Throughout the paper, we will restrict the descriptions synergistic and antagonistic to non–sign epistasis.

In [5] it was found that the prevalence of the three categories of epistasis undergoes significant change along an adaptive walk, with sign epistasis increasing in frequency as the walk progresses, and antagonistic epistasis decreasing relative to sign epistasis and marginally decreasing relative to synergistic epistasis. The authors discuss the phenomenon in some generality and analyze empirical examples. However, in their explanation, the authors confine themselves to NK models [14], [15], and their arguments are dependent of the details of how NK models are defined and constructed.

The goal of this study is to investigate this phenomenon among a more general class of fitness landscapes, and provide an explanation independent of model specific assumptions. We appreciate that the classical models, including the NK model are valuable for testing ideas. However, explanations independent of structural assumptions on the landscapes are desirable, especially since it is unclear how relevant the classical models are for empirical fitness landscapes.

## Results

We consider two types of fitness landscapes in our simulations: NK models and “Rough Mt. Fuji” models [7], [16], [17]. The precise definition of both types of landscapes are found in Materials and Methods. Briefly, the fitnesses of genotypes in an NK landscape are determined by the fitness contribution of each locus. The fitness contribution of each locus is a stochastic function of its own state plus the state of K other loci which are fixed in advance. When K = 0, the landscape is purely multiplicative (or additive, depending on our choice of model), and (in the multiplicative case) would have no epistasis. At the other extreme, when K = L−1, the fitnesses of genotypes are mutually independent, leading to abundant epistasis. (The NK model is sometimes denoted the “LK model”. We will use the term NK model, although we consider L loci.)

The so called Rough Mt. Fuji models are constructed by starting with a purely additive or multiplicative model, where each allele contributes a fixed, equal amount, independent of background. The determinate fitnesses obtained this way are then perturbed by random noise. See Materials and Methods for further details on the construction of Rough Mt. Fuji landscapes, as well as some comments about multiplicative and additive assumptions. In this study we confine ourselves to additive Rough Mt. Fuji landscapes, though we note that simulations performed with multiplicative Rough Mt. Fuji models (and which are not reported in this study) support the conclusions below. We fine tune the relative magnitudes of random noise and fixed additive contribution with a parameter, thereby allowing us to vary Rough Mt. Fuji landscapes in a manner analogous to varying NK models with the choice of K.

We will be concerned with the properties of adaptive walks in our fitness landscapes. We will assume the asymptotic condition of Strong–Selection–Weak–Mutation (SSWM for short) [18]–[20], s. It is assumed that the evolving population remains genetically monomorphic outside of very short time intervals, during which a new beneficial mutation sweeps to fixation. Given a genotype 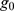, population genetics theory shows that if the selection coefficients of the fitter mutational neighbors 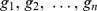 of 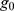 are 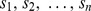, respectively, then the probability of 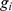 going to fixation is
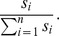
(It should be noted that we are sweeping under the rug the fact that strictly speaking this formula is appropriate only when the magnitudes of the second or higher powers of the 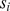 are negligible.) For more background about the SSWM assumption, as well as the fixation probability described, see [21].

An adaptive walk, then, can be viewed as a stochastic path in a fitness landscape, starting at an initial genotype and ending at a genotype with locally maximal fitness. For every two steps in such a walk, three genotypes are traversed, which can be denoted, in order, 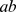, 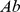, and 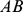. (Note that we are no longer assuming the minimality of 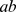 as was done in Figure 1.) These genotypes are complemented by 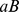, and the type and magnitude of epistasis for the quadruple can be determined by their fitnesses. Note that the configuration in Figure 1 D has no relevance for adaptive walks, and makes no appearance in subsequent calculations.

In [5], it was noted that the relative frequencies of sign, antagonistic, and synergistic epistasis varied along adaptive walks. Our aim is to explore this phenomenon more closely. What are the relative frequencies of sign, antagonistic, and synergistic epistasis?

In our notation, we assume that three genotypes ab, Ab and AB are traversed in some adaptive walk, so that
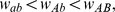
and consequently 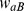 determines the type of epistasis (again, we do not assume that 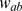 is minimal). These assumptions hold for the remainder of this paper. The possibilities are that 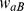 is ranked first, second, third or fourth in terms of fitness relative to the other three genotypes. *When ranked first or fourth, the quadruple has sign epistasis, and not so when ranked second or third*. This fact will be used repeatedly.

We start with a preliminary observation. In the special case where fitnesses of mutational neighbors are identically and independently distributed, such as in an NK landscape with 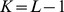, and where the genotypes are chosen randomly, the probabilities that 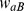 is ranked first, second, third or fourth are readily calculated. Indeed, the probabilities are equal, since the fitness of a paticular genotype is independent of mutational neighbors. Consequently sign epistasis occurs with frequency 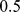.

Similarly, consider a randomly chosen quadruple but in la andscape where the fitness of mutational neighbors are correlated, as in NK landscapes with 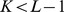. Then we expect the frequency of sign epistasis to decrease relative to the case of uncorrelated fitness. This expectation is confirmed by simulations, the results of which are found in Text S1. The parameter 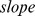 in the Rough Mt. Fuji models is positively associated with correlation between mutational neighbors. (See Text S1) The simulation results thus confirm the expectation of lower sign epistasis in landscapes with correlated mutational neighbors.

The results of our simulations confirm [5], namely that the further one is along an adaptive walk, the larger the frequency of sign epistasis and the smaller the amount of antagonistic epistasis relative to synergistic epistasis. Significantly, a similar evolution of relative frequencies occurs in the Rough Mt. Fuji landscapes. It is clear that a more general explanation for this phenomenon is desirable, since Rough Mt. Fuji fitness landscapes are not defined in terms of locus–by–locus fitness contributions.

We hypothesize that the observed evolution of epistasis along adaptive walks is merely the familiar statistical phenomenon of regression to the mean. This explanation was suggested in [5] as well. However, the authors' arguments are restricted to the details of the NK model. We offer here a simpler and more general explanation.

We begin with an intuitive explanation for the phenomenon we seek to explain. This will be followed by evidence from simulations that support our argument. We consider the type of epistasis that would be found with respect to a quadruple of genotypes 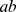, 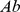, 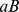, and 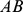, where 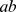, 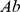, and 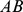 form three subsequent genotypes in an adaptive walk.

Informally, the following extreme example will clarify the picture somewhat. Suppose that 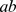 belongs to the highest fitness percentile among genotypes in the fitness landscape. For uncorrelated fitness, the expected frequency of sign epistasis would be at least 99 percent. Indeed, one would get 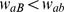 in 99 percent of the cases. Similarly, for correlated fitness one would many times get 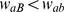 as well, provided there is sufficiently much noise in the landscape. This is because a mean regression effect will tend to “pull” the fitness of 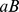 below 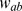, since 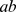 belongs to the highest fitness percentile.

After the informal example, we now go over the different possibilities for the quadruple of genotypes in some detail. We will compare low and high fitness of 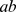 with the “null” condition where 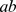 is randomly chosen. If we impose the condition that 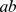 has lower fitness relative to the mean fitness of the landscape, then it is likely that 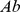 and 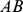 will have lower fitness than would have been expected if 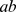 had been randomly chosen (unless the fitness landscape is uncorrelated, of course), though the likelihood of large jumps in the adaptive walk may return 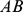 to more typical fitness levels. To the extent 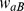 is determined by a stochastic component independent of 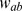, 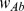, and 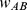, mean regression implies that it is more likely that 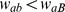 than in the case where 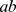 is randomly chosen without condition from the fitness landscape. Note that the imposed condition of relatively low 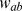 biases the probability toward non-sign epistasis relative to the “null” condition. Furthermore, within the region of non–sign epistasis, the bias toward 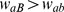 relative in the null situation results in a higher probability that
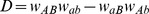
is negative, leading to a bias toward antagonistic epistasis.

Conversely, when an adaptive walk reaches 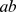 after a number of steps, and continues to 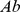 followed by 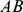, it is highly likely 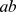, 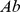, and 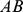 have high fitness relative to the mean fitness of the fitness landscape. To the extent that 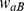 is determined by a stochastic component independent of 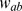, 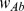, and 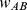, mean regression implies that 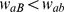 is more likely than would be the case when 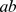 is randomly chosen without condition. Furthermore, within the interval of non–sign epistasis, the quantity 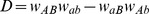 is biased upward toward positive values, thus leading to a higher proportion of synergistic epistasis to antagonistic epistasis. We conclude that the changing balance of types of epistasis along an adaptive walk is not due to any intrinsic feature of adaptive walks per se, but rather the result of traversing from lower to higher fitnesses. Late stage adaptive walks are “walking along a ridge”, implying more sign epistasis. In summary, the pattern of changing epistasis along an adaptive walk is driven by mean regression due to the fitnesses of 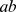, 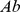, and 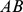 and the uncorrelated component of the fitness of 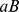.

**Figure 3:**
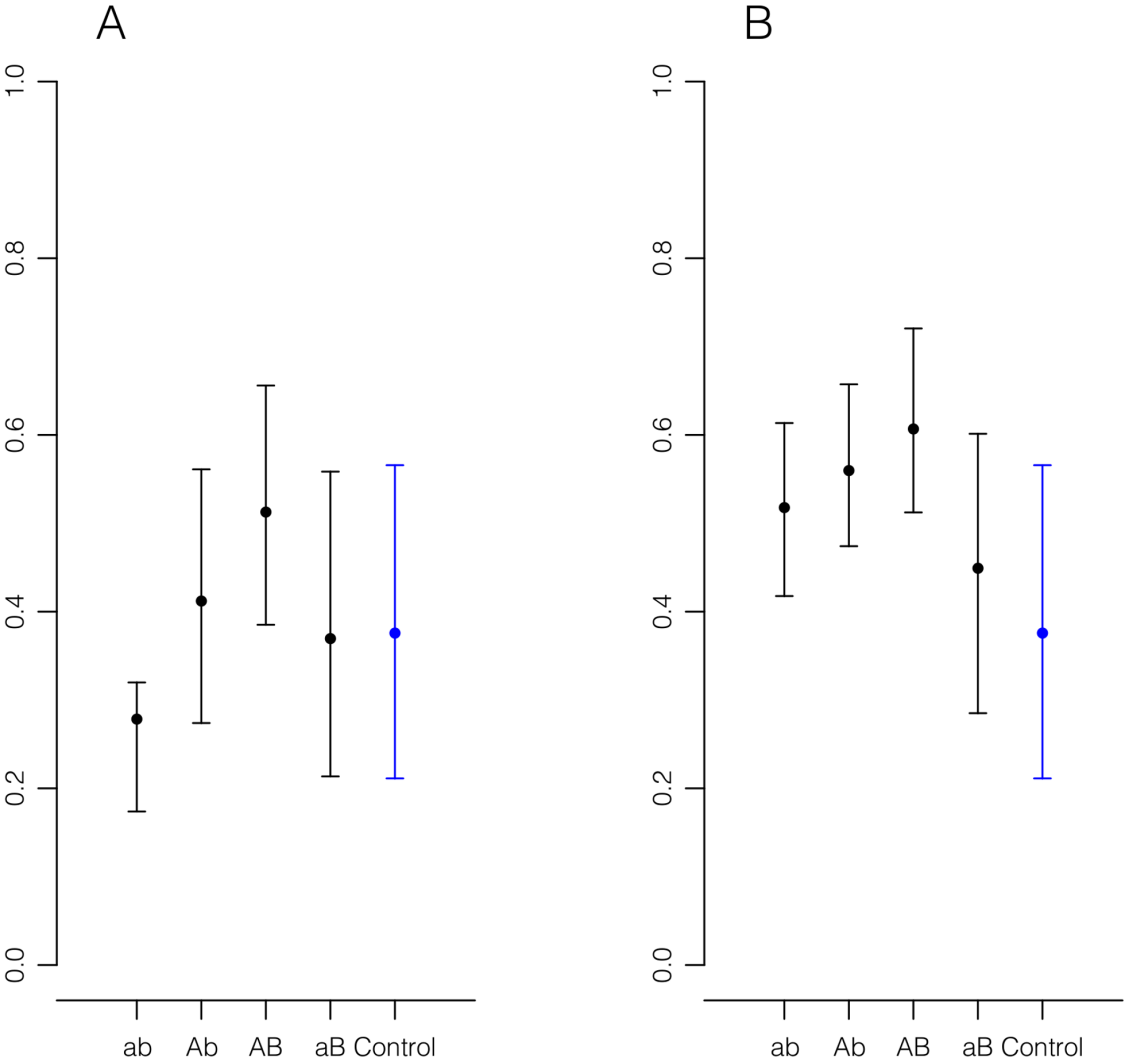
1000 adaptive walks simulated on NK landscapes with N = 15 and K = 10. For each walk, the starting genotype 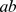 was randomly drawn to have relatively low fitness (see Text S1 for details). **A.** Intervals covering fitnesses between the 2.5 and the 97.5 percentiles are shown for the first (ab), second (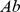), and third (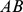) genotypes in randomly generated adaptive walks, with dots indicating the medians. The genotype 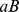 is the remaining genotype in the quadruple as shown in Figure 1. The blue “Control” interval corresponds to randomly selected genotypes. The skew visible in the ab interval is due to the fact that the initial genotype of a fitness walk is drawn from a lower tail distribution. **B.** Intervals for the fourth, fifth, and sixth genotypes in randomly generated adaptive walks. The increased fitness of the aB genotypes in B relative to that of A is due to the fact that 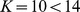, and thus there is some correlation between neighboring genotypes. In both diagrams, the dependency of sign epistasis on regression to the mean is apparent.

We remark that our simulations of adaptive walks reveal an interesting asymmetry between 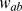 being far below, and far above the mean (see Figure 3). Indeed, the quantity 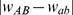 tends to be relatively large for very low 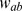 and relatively small for very high 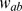. In particular, the asymmetry helps explain why the frequency of sign epistasis depends on the fitness of 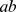 for the landscapes we simulated. One can ask how general the observed asymmetry is. Some caution is necessary depending on the fitness distribution, and it would be interesting to further explore the problem.

**Figure 4:**
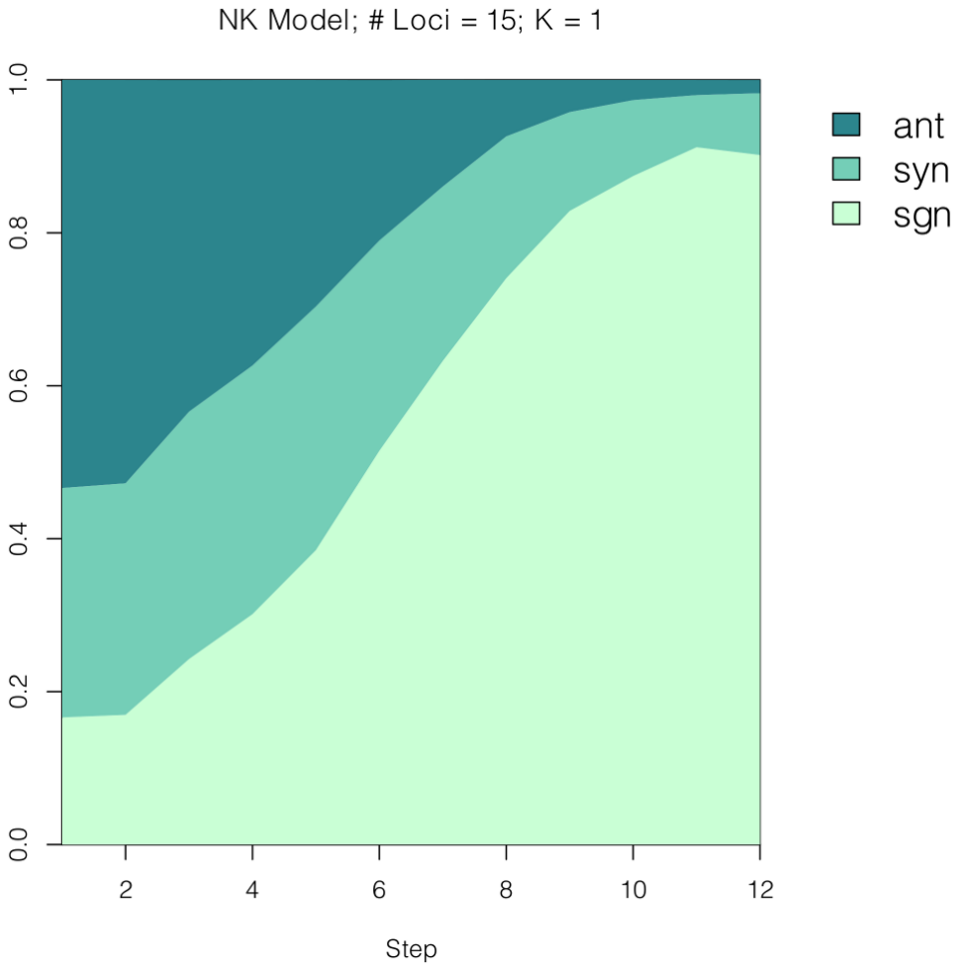
According our simulations, the patterns of epistasis change along adaptive walks as displayed. The graph depicts NK landscapes with parameters 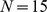 and 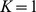.

Figure 4 depicts the patterns of epistasis along adaptive walks. The patterns agree with our intuitive description. The figure concerns the NK landscape with parameters 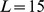 and 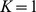. See Materials and Methods for a complete description of our simulations of adaptive walks.

**Figure 5:**
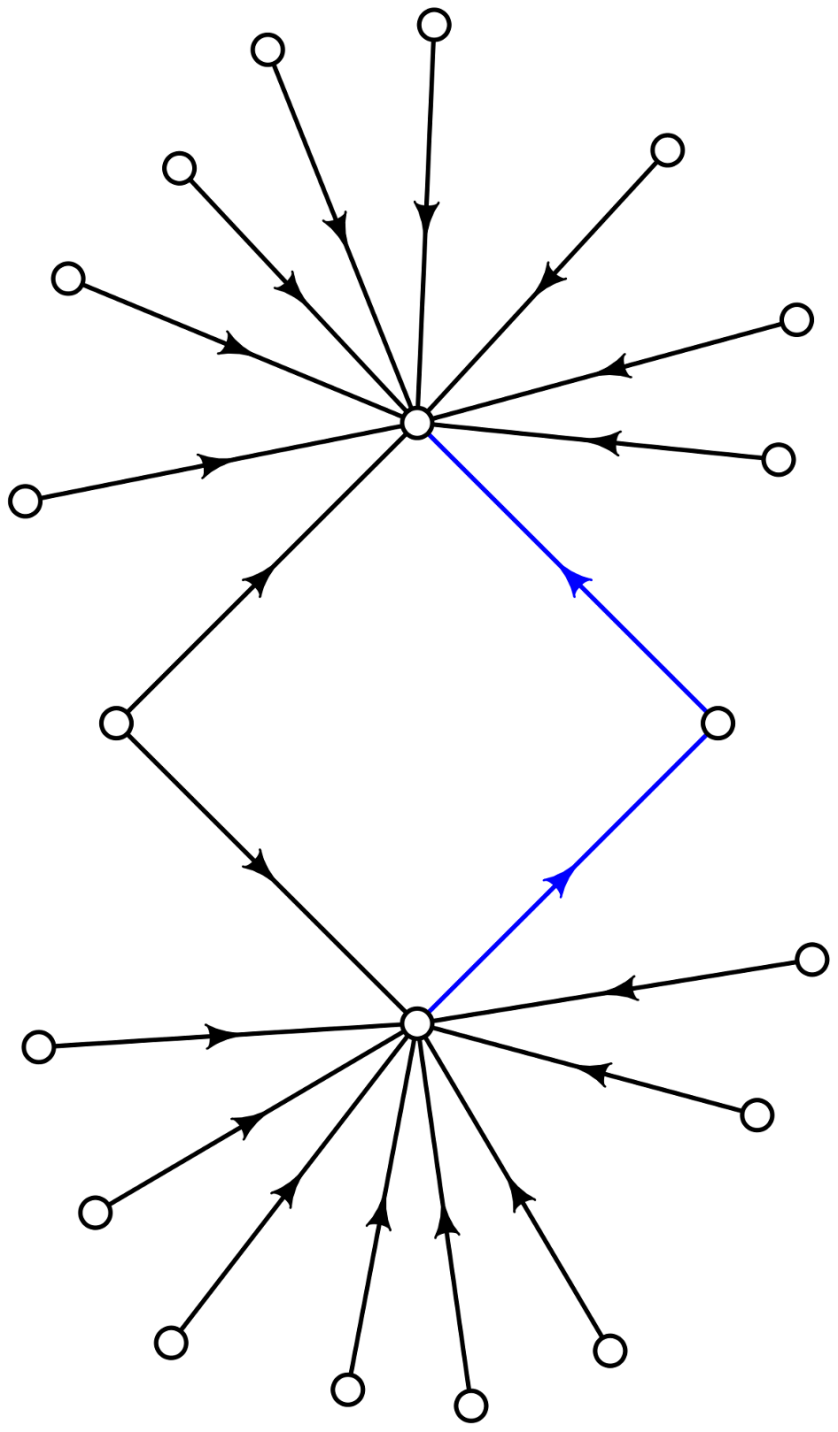
Assume that the adaptive steps, colored blue, connect three genotypes with relavatively high fitness. Most connecting arrows point toward the starting point, as well as the end point of the adaptive steps. Note that due to the high fitness of the genotypes along the adaptive walk, the arrows emanating from the fourth genotype in the quadruple are more likely to point outward. The result in such a case is sign epistasis.

The case of high 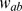 is illustrated somewhat crudely in Figure 5. The blue arrows form part of an adaptive walk, and the three vertices they connect correspond to 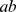, 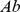, and 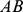 above. If we assume that 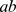 has higher than average fitness, then when the fitness of genotype 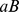 has an uncorrelated component there is a bias toward 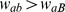, leading to sign epistasis.

**Figure 6:**
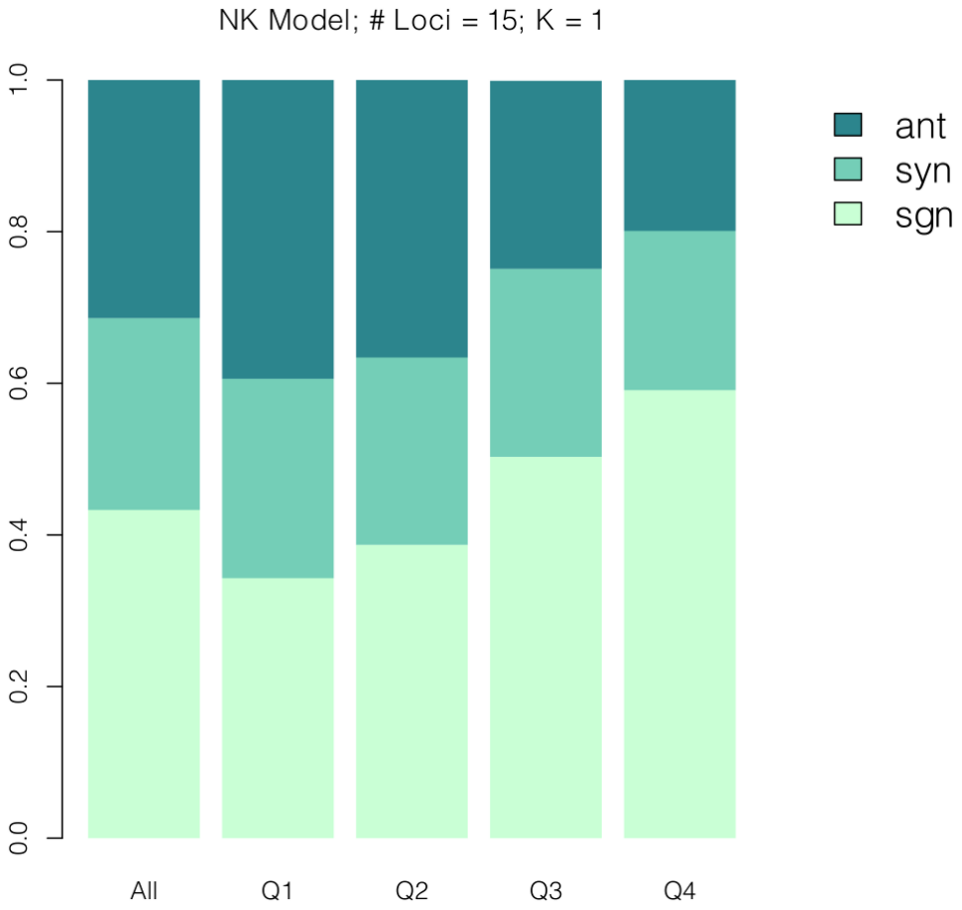
Random quadruples were sampled in a stratified fashion, where *w_ab_* belongs to the specified fitness quartile. The frequencies of sign, antagonistic, and synergistic epistasis should change their relative proportions from the lowest quartile to the highest quartile as they do along an adaptive walk.

We buttressed our intuitive argument above by examining the results of simulated fitness landscapes and adaptive walks. The results of these simulations are attached as a supplement to this article. If our explanation above is correct, two results should emerge from our simulations. One, if random quadruples of genotypes as shown in Figure 1 are sampled in a stratified fashion from different fitness quartiles of the landscape, then the frequencies of sign, antagonistic, and synergistic epistasis should change their relative proportions from the lowest quartile to the highest quartile as they do along an adaptive walk. They do, as can be seen in Figure 6 and in Text S1. (To clarify, we sampled 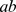 so that 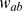 belongs to the specified quartile. We did not impose any conditions on the genotypes 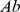 and 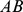 beyond 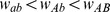).

Two, if we simulate adaptive walks under the condition of equal probabilities among all mutational neighbors, the rate at which fitness increases should be slowed, and therefore the frequencies of types of epistasis should change at a slower pace than they do in a weighted probability model. They do, as can be discerned by comparing the figures with equally weighted probabilities, to the figures with probabilities weighted according to the SSWM model (see Text S1).

Further support for our proposed explanation was obtained by simulating 1000 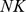 landscapes with 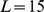 and 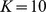. The result, summarized in Figure 3, confirm our assertions.

For each landscape, a genotype with relatively low fitness was chosen as the initial genotype of an adaptive walk (see Text S1 for details). Figure 3 summarizes the important features of the results of the simulations. In caption A, 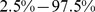 percentile intervals are shown for the first(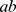), second(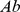), and third(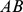) genotype of the adaptive walk. The fourth interval corresponds to the complementary genotype 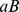. The ranges of the intervals show a bias toward non-sign epistasis. The blue “control” interval corresponds to randomly selected genotypes.

Conversely, in caption B, 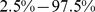 percentile intervals are shown for the fourth(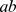), fifth(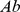), and sixth(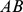) genotypes visited on an adaptive walk. Again, the fourth interval corresponds to 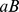. In this case, the bias is toward high frequency of sign epistasis.

**Figure 7:**
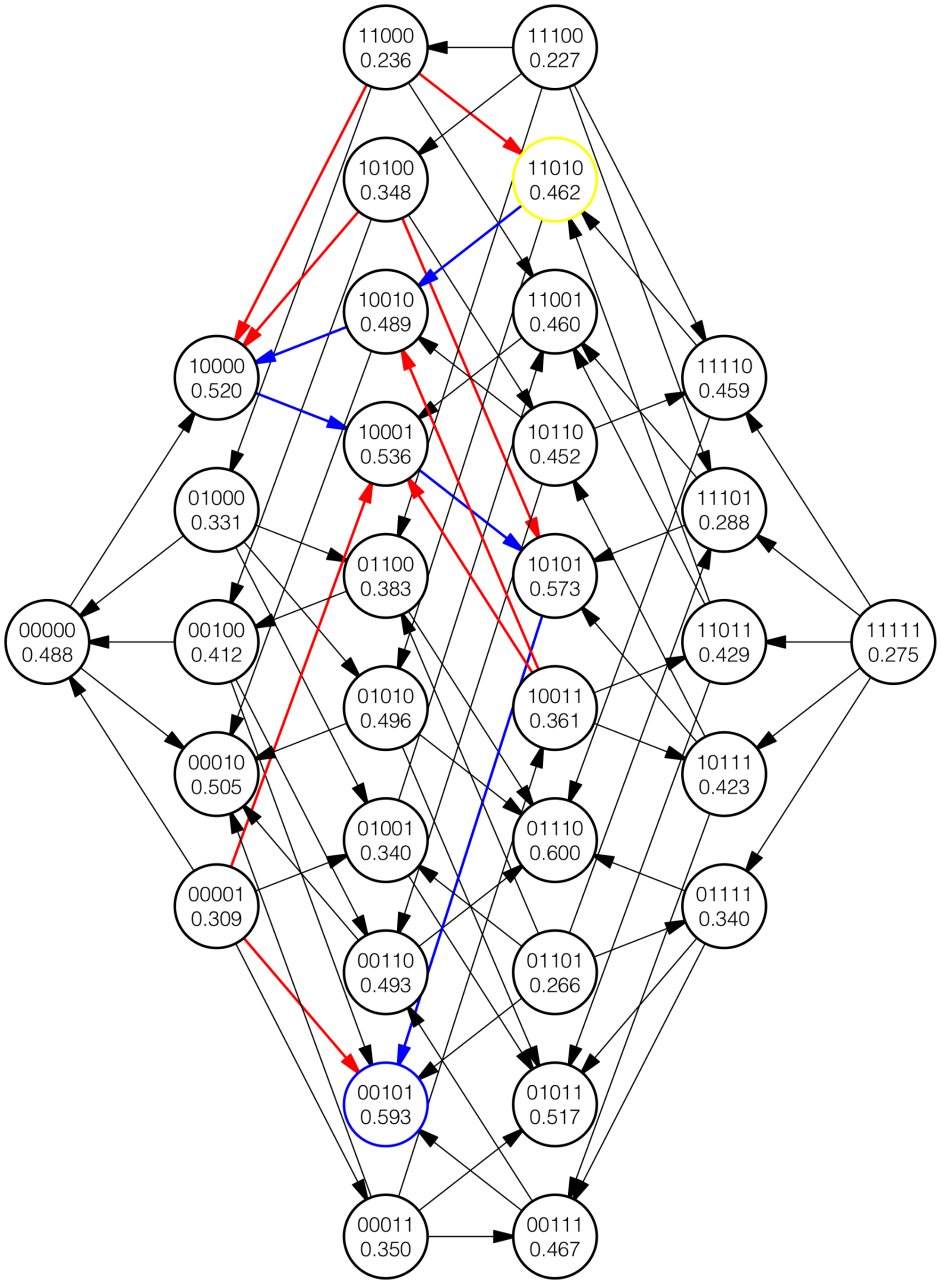
A depiction of the fourth (yellow), fifth, sixth, seventh, and eighth genotype of an adaptive walk in an NK landscape, with *N* = 15 and *K* = 10. Only loci affected by mutation during the five adaptive steps are shown in the genotype labels, and the genotypes shown are restricted to those that differ from the initial genotype only at the five affected loci. The fitness of each genotype is also shown. The adaptive walk is colored blue, while the opposing arrows in each quadruple are colored red. Note the dominance of sign epistasis along the adaptive walk. The ridge-like quality of the adaptive walk is clear from the high proportion of “in” arrows emanating from the evolved genotypes.

**Figure 8:**
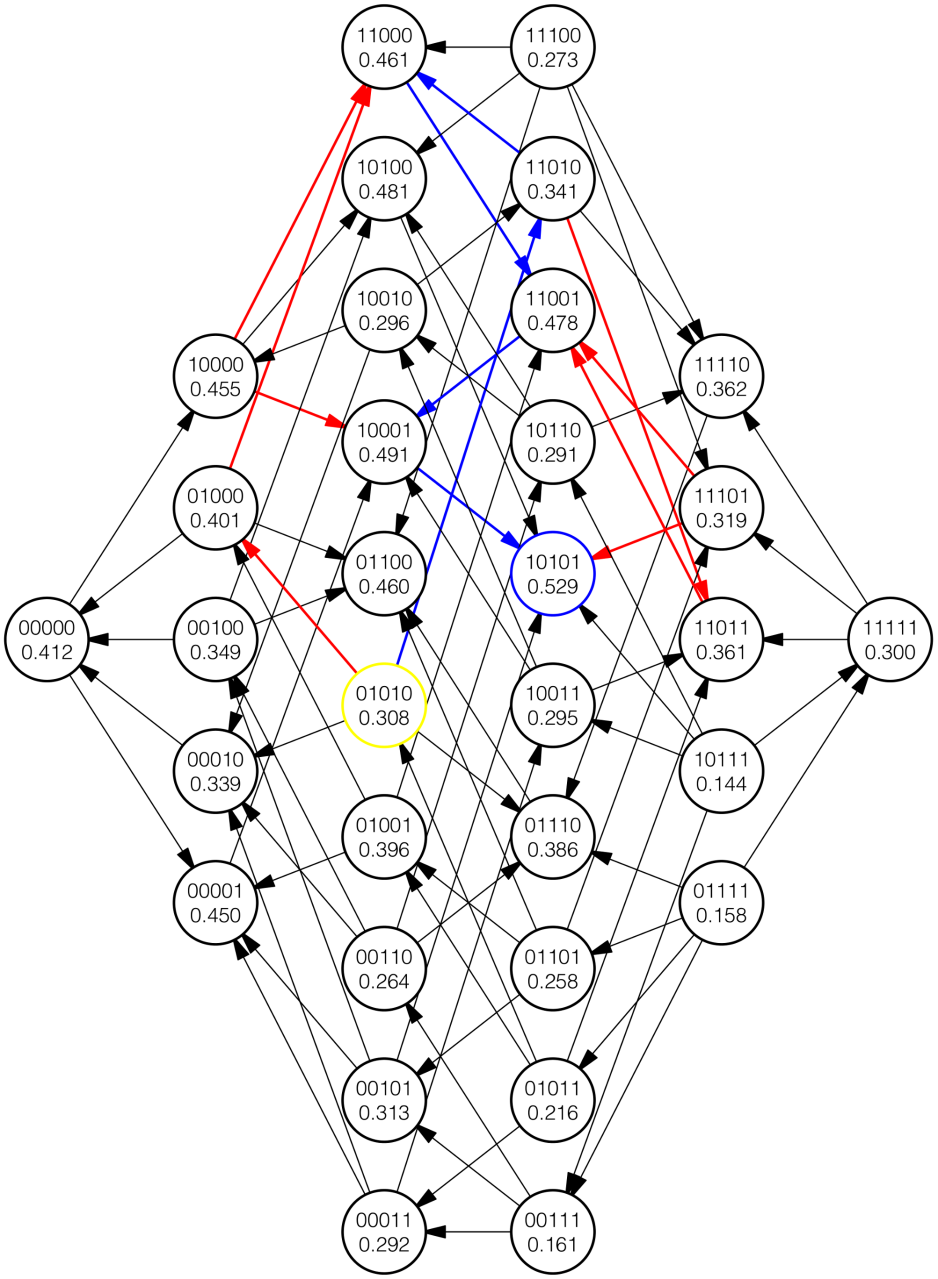
A depiction with a description analogous to Figure 7 but in contrast, the yellow colored genotype is the initial genotype of the adaptive walk. Note the lower frequency of sign epistasis along the walk as compared to Figure 7.

In both cases, the role of mean regression in driving the nature of epistasis along adaptive walks is apparent. Figures 7 and 8 represent partial views of one simulation as described above. Even here, the bias toward or away from sign epistasis depending on the stage of the adaptive walk is apparent.

We have compared equal weights, and adaptive walks under the SSWM assumption. For more background and results regarding lengths of walks, we refer to [22], [23] for equal weights, and [21] for the SSWM case.

As a final remark, the study of epistasis as described was restricted to pairwise interactions. It would be interesting to extend the study to higher order interaction, and for instance to consider shapes as defined in the geometric theory of gene interactions [2], [24].

### Empirical support and applications

As mentioned in the introduction, empirical data seem to support the “mean regression” hypothesis exposited herein. We add further support with the following empirical results from investigations of the TEM-family of 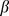-lactamases [25]. The TEM-enzymes are associated with resistance to several 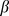-lactame antibiotics, including penicillins. TEM beta-lactamases have been found in Escherichia coli, Klebsiella pneumoniae and other Gram-negative bacteria. TEM-1 is considered the wild-type, and approximately 200 mutant variants have been found clinically, (see e.g. the record from the Lahey Clinic http://www.lahey.org/Studies/temtable.asp).

For the 4-tuple mutant TEM-85 (L15F, R164S, E240K, T265M) the two fitness landscapes defined by Cefotaxime and Ceftazidime had mutational trajectories (i.e. adapative walks) from TEM-1 to TEM-85. For Cefotaxime there were three trajectories to TEM-85, and for Ceftazidime one trajectory. We calculated the epistasis in the last two steps, as well as in the first two steps, of the four trajectories. Fitness differences of mutational neighbors were not always statistically significant in the study, resulting in cases of “possible” sign epistasis. The results for the last two steps were two cases of sign epistasis, and two cases of possible sign epistasis. The results for the first two steps were two cases of possible sign epistasis, and two cases of no epistasis. These findings seem to support our hypothesis, though we must refrain from drawing any sweeping conclusions based on a small data set.

Generally speaking, there are two types of empirical studies of evolution, direct and indirect. A direct study is concerned with an evolving population, where mutations are observed as they occur. Examples of this are a population evolved in a laboratory or the stages of an HIV infection due to drug resistance conferring mutations. The second type of study is indirect. An investigator attempts to create a catalog of genotypes with the potential of being part of an adaptive walk. As an example, a strain of bacteria that is highly resistant to a particular antibiotic treatment may differ from the wild-type by 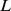 amino acid substitutions in a relevant enzyme. The investigator in an indirect study will attempt to produce and study all 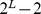 intermediate mutational stages. It is non-trivial to relate direct and indirect studies. One wishes to infer the fitness landscape from an evolving population. Conversely, one would like to predict evolution from indirect studies. As observed in [5], epistasis may influence path choice for evolving populations, and path choice has an impact on epistasis. Consequently, it may be difficult to infer the fitness landscape from a direct study.

As for the converse, it may seem straightforward to predict evolution from a fitness landscape. However, a practical difficulty arises; namely, the information one has in an indirect study is often restricted to the fitness *rankings* of the genotypes, with no quantitative measurements of fitness. Consequently, one has very little knowledge of the probabilities of evolutionary trajectories, even if the fitness graph is known.

At issue here is the fact that examining epistasis in fitness graphs and evolving populations may lead to results which seem at odds. It is *a priori* not clear if patterns of epistasis along adaptive walks are easily predicted from fitness graphs. In addition to being used for confirming the robusticity of our results, we included the equally weighted adaptive walks (see Text S1) to reflect the point of view of the results of an indirect study, where only the fitness rankings of the genotypes in the landscape are discovered, and thus there is no *a priori* knowledge of the appropriate weights to be assigned to the various paths evolution may follow. The pattern of epistasis was broadly held across the two classes of fitness landscapes considered here, across a range of parameters for these landscapes, and across the weighted versus the unweighted versions discussed above. (The main difference we could find was pace in which proportions of epistasis changed, which is easily explained by the fact that the rate of fitness increase is slower in the equally weighted walk.) If we consider the equally weighted case as corresponding to indirect studies, and the weighted case to direct studies, then it is interesting to note while the rate of change of the proportions varies, the general pattern does not. Naturally it would be interesting to further investigate the relation between direct and indirect studies of adaptation.

## Discussion

The nature of epistasis varies along an adaptive walk. This observation has been made in simulations, and has support in some empirical studies. We have argued that mean regression is a simple and general explanation for this phenomenon. We support this explanation with simulations carried out on two classes of fitness landscapes, with varying parameters. While our simulations were restricted to two classes, our argument should extend to any fitness landscape where genotypes vary to any degree independently to each other.

We considered two types of adaptive walks; those with probability weight corresponding to those used in the SSWM model, and those with equal probability weights. The similarity of the results suggests that the pattern of epistasis found along an adaptive walk is not a result of any specific property of adaptive walks generated according to the SSWM model. This result is also relevant for relating direct and indirect studies as defined above.

Further support for our assertion was obtained by sampling genotypic quadruples of mutational neighbors from simulated fitness landscapes at different fitness quartiles. The resulting pattern of increasing sign epistasis and decreasing antagonistic to synergistic ratio at higher fitnesses relative to lower fitnesses reinforces our assertion that the same phenomenon seen along adaptive walks depends on mean regression, and does not depend on any intrinsic properties of adaptive walks per se.

Our main observation has important consequences for interpretations of empirical data. Consider any fitness landscape where there is a well defined wild-type, and some beneficial single mutants. For instance, the fitness landscape may be associated with antimicrobial drug resistance. Some recent papers consider prevalence of sign epistasis, and related questions for such landscapes, where the wild-type is used as a starting point (for a survey article, see e.g. [13]) Our result demonstrate that there are two factors that influence the prevalence of sign epistasis [26]. The first is the degree of additivity in the landscape. The second is the fitness of the wild-type. Ideally, a study should therefore estimate wild-type fitness as well as additivity in the landscape. Roughly, one can estimate wild-type fitness from the proportion of single mutants which are more fit than the wild-type among all mutational neighbors of the wild-type (see e.g. [Crona et al., 2013] for more comments).

We have argued that our main observation holds for empirical fitness landscapes. Most aspects of adaptation are sensitive to epistasis. In particular, a serious analysis of recombination requires a fine-scaled understanding of epistasis. It would be interesting to explore recombination in light of our findings.

## Materials and Methods

Throughout this study, loci were considered to be bi–allelic, with alleles 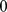 and 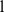 for each locus. All of the fitness landscapes had 15 loci.

The NK model is classical. The so–called Rough Mt. Fuji model has been explored.

Some of the features of our fitness landscapes were peculiar for this study, so we will summarize briefly in this section how they were constructed.

For the NK fitness landscapes, the contribution of each locus is a function of the allele at the locus itself as well as the alleles at 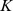 randomly chosen additional loci, or
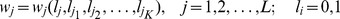
The fitness of a particular genotype 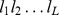 is then the geometric mean of the individual loci contributions:
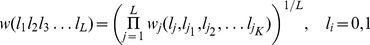
(1)


For each of the possible values of 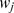, we sampled independently from a uniform distribution over the interval 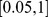. The 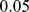 floor was used to prevent overly large fitness coefficients.

Since calculating the fitness of each genotype in an NK landscape proved computationally time–consuming, we determined the fitness quartiles theoretically as follows. Since the logarithm of the right hand side of (1) is the mean of 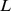 identically distributed independent variables, by way of central limit theorem we approximated the distribution of fitnesses using a Gaussian distribution. The quartile boundaries were then determined from this approximation. Some test simulations showed this to be a reasonably accurate approximation.

To explore fully the changing nature of epistasis along an adaptive walk, for the initial genotype we sampled from genotypes with fitness below the mean minus 1.5 standard deviations according to the theoretical approximation. This corresponds (again, theoretically) to the 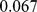 quantile of the distribution.

Our Rough Mt. Fuji fitness landscapes were constructed in the spirit of their namesakes in the wider literature. At first, each genotype is assigned a deterministic fitness component given as follows:
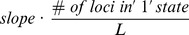
where *slope* is a pre–determined fixed parameter. To each of these deterministic values a random value drawn from a uniform distribution on 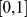 is added.
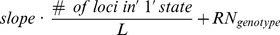



Finally, we applied a linear transformation making the minimum and maximum fitnesses 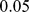 and 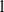 respectively. Note that by our construction the “expected” fitness difference between the genotypes 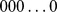 and 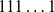 will be 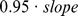. The parameter *slope* determined the relative contributions of the deterministic component and the noise component in the landscape, with high values of *slope* implying a low ratio of noise component to deterministic component.

Since the computation of empirical quantiles was feasible for Rough Mt. Fuji landscapes, we used them for determining quartile boundaries and selecting initial genotypes. The latter were selected from those genotypes with fitnesses among the bottom 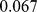, as they were chosen in the 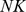 landscape case, but in this case using the empirical quantile rather than the theoretical quantile.

As for the simulations, it should be pointed out that confidence intervals and issues with statistical power were ignored in this article. For each set of parameters, we simulated 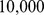 fitness landscapes with an adaptive walk. It can be seen from the figures in Text S1 that for most types of landscapes the number of adaptive walks which evolve to an 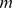
th genotype before hitting a local optimum decreases quite significantly with 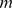 after approximately the four steps. Naturally, the low number of adaptive walks which attain higher steps may raise concerns of statistical power. Nevertheless, despite this possible shortcoming, we feel that the general pattern is clear enough.

Let us also remark that our choices of multiplicative or additive scales were made mostly for convenience throughout the article. Our main observations are independent of such choices.

All simulations were coded in the programming language R [27], and we used the R package [28].

## Supporting Information

Text S1 Supplementary information.
(PDF)
