# Supplementary material for: The Changing Geometry of a Fitness Landscape Along an Adaptive Walk
Source: PLoS Comput Biol. 2014 May 22;10(5):e1003520. doi: 10.1371/journal.pcbi.1003520 (PMC4031059; doi:10.1371/journal.pcbi.1003520)
Supplement: Text S1 — Supplementary information. (PDF) [file pcbi.1003520.s001.pdf]

SUPPLEMENTARY INFORMATION

Table Key

The results of our simulations are summarized in 24 diagrams. In each diagram, the relative proportions are given for antagonistic, synergistic, and sign epistasis as defined in the main article. The proportions represented are among those quadruples which had epistasis. The first figures are the results of the "stratified" sampling as described in the main text. The proportions represented in the "All" column are the result of 10,000 simulations. Each quartile column, labeled Q1 through Q4 is the result of 2500 simulations each. For the second set of figures, 10,000 fitness landscapes are simulated (figures with the equal weight assumptions are labelled accordingly, and the remaining figures concern the SSWM assumption). In each, an initial genotype is selected in the manner described in Materials and Methods. An adaptive walk is then simulated. Upon reaching the third genotype in the walk, the epistasis is calculated for that genotype and the previous two. This first calculation corresponds to Step 1 in the horizontal axis. The relative proportions of subsequent calculations of epistasis are recorded in Step 2, Step 3, etc. The meaning of the parameters used in generating the fitness landscapes is discussed in Materials and Methods.

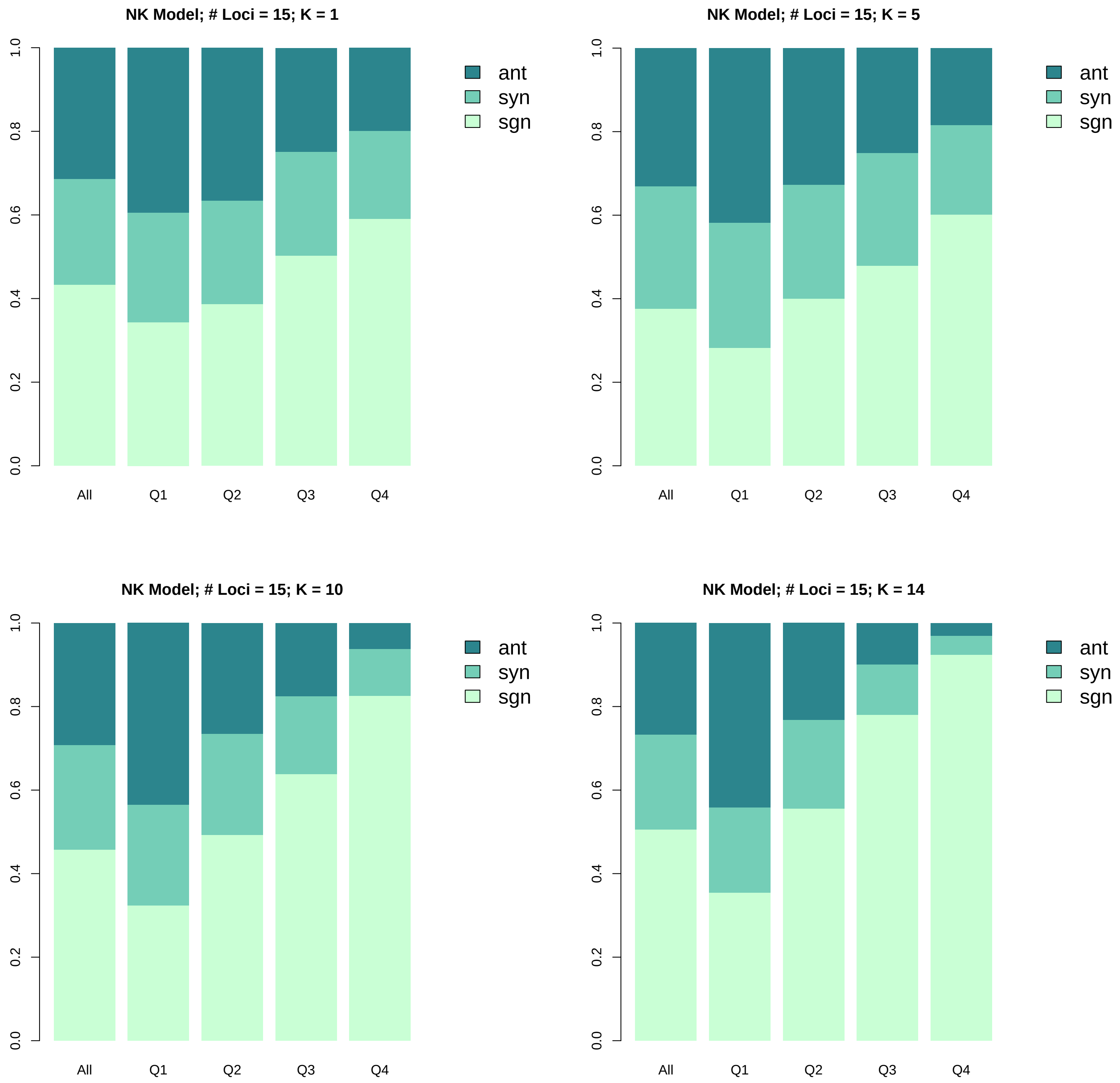
NK Model; # Loci = 15; K = 1
NK Model; # Loci = 15; K = 5
NK Model; # Loci = 15; K = 10
NK Model; # Loci = 15; K = 14
0.0
0.2
0.4
0.6
0.8
1.0
All
Q1
Q2
Q3
Q4
ant
syn
sgn

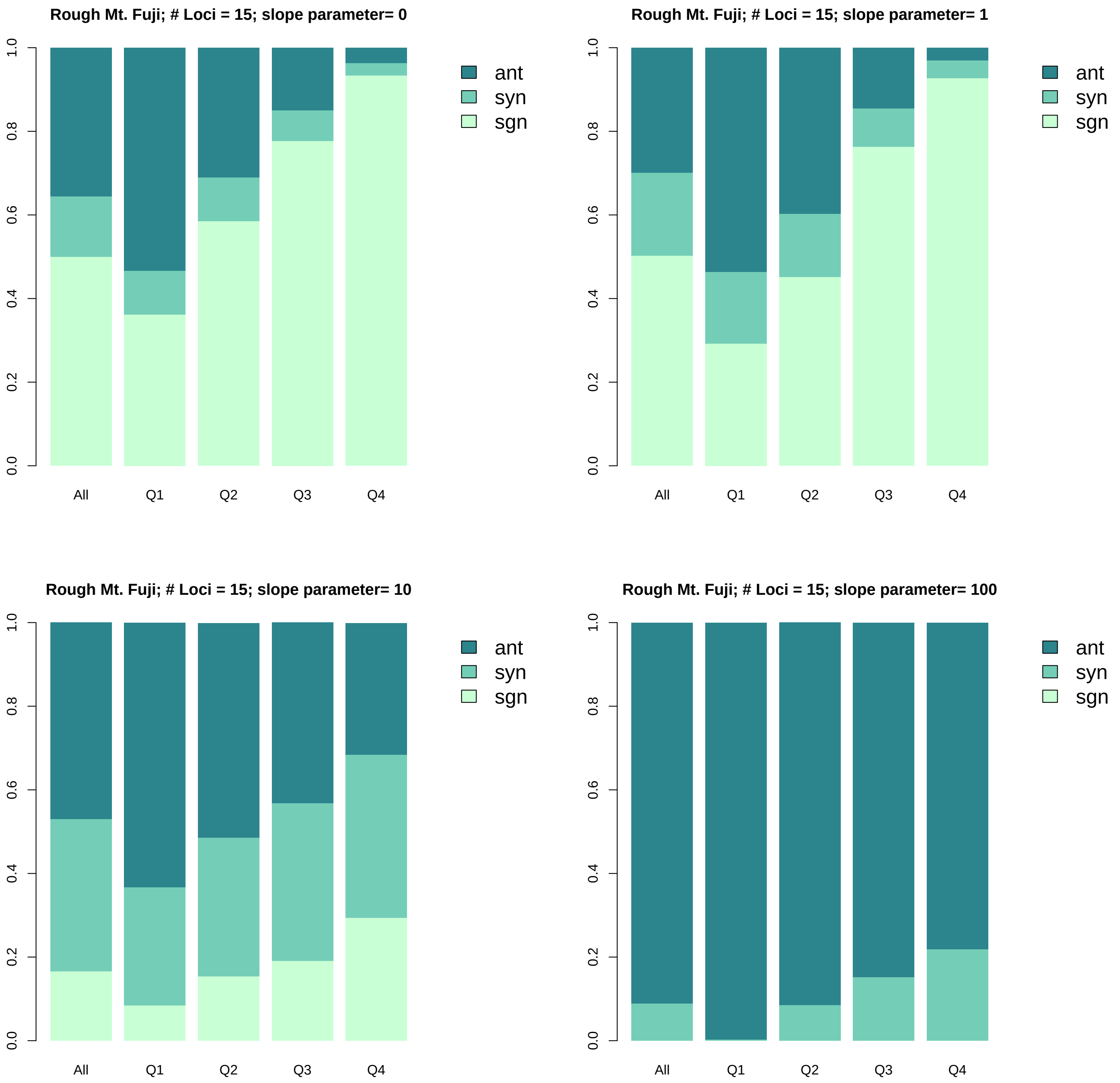
Rough Mt. Fuji; # Loci = 15; slope parameter= 0
Rough Mt. Fuji; # Loci = 15; slope parameter= 1
Rough Mt. Fuji; # Loci = 15; slope parameter= 10
Rough Mt. Fuji; # Loci = 15; slope parameter= 100
0.0
0.2
0.4
0.6
0.8
1.0
All
Q1
Q2
Q3
Q4
ant
syn
sgn

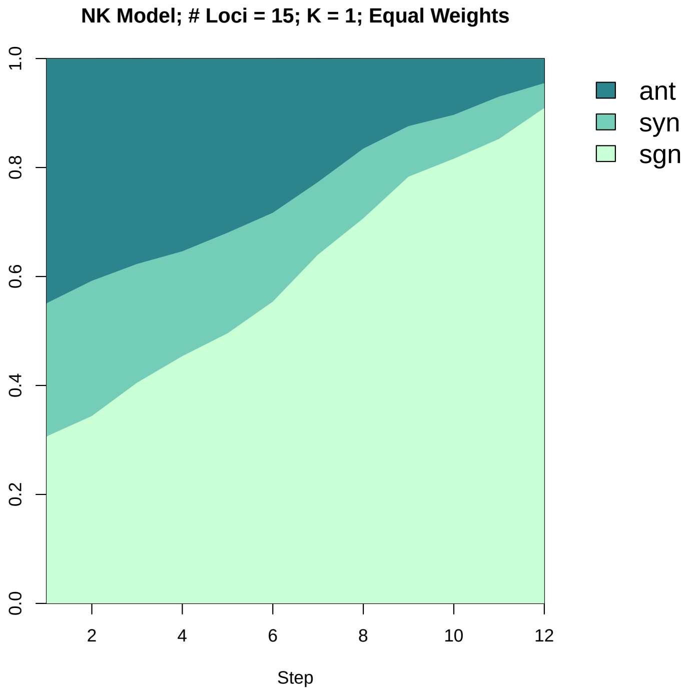
NK Model; # Loci = 15; K = 1; Equal Weights
0.0
0.2
0.4
0.6
0.8
1.0
2
4
6
8
10
12
Step
ant
syn
sgn

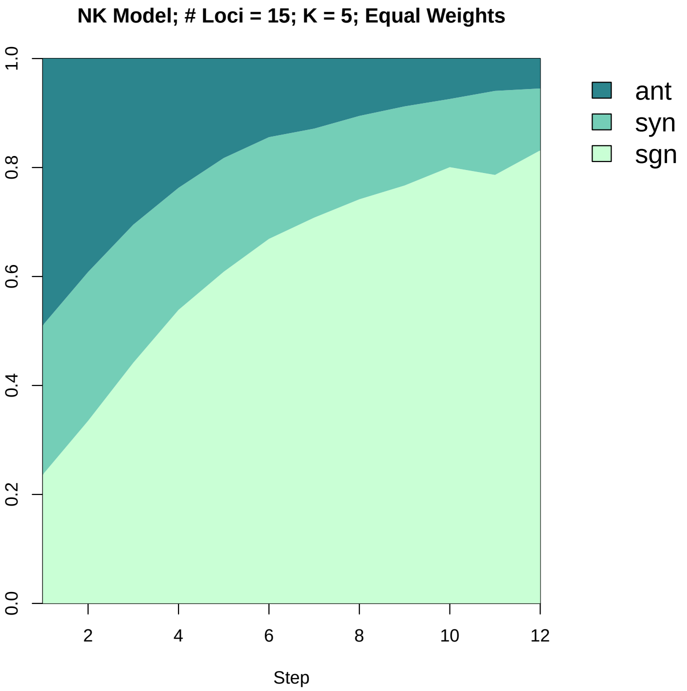
NK Model; # Loci = 15; K = 5; Equal Weights
0.0
0.2
0.4
0.6
0.8
1.0
2
4
6
8
10
12
Step
ant
syn
sgn

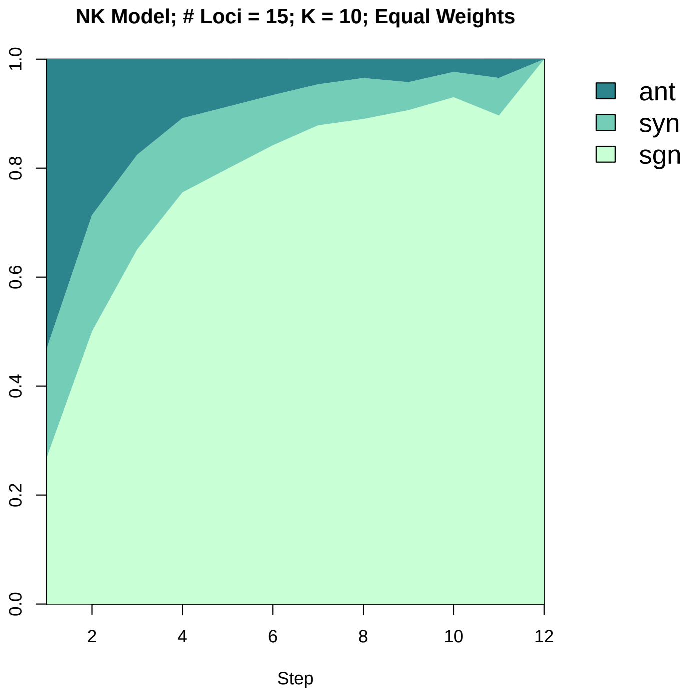
NK Model; # Loci = 15; K = 10; Equal Weights
0.0
0.2
0.4
0.6
0.8
1.0
2
4
6
8
10
12
Step
ant
syn
sgn

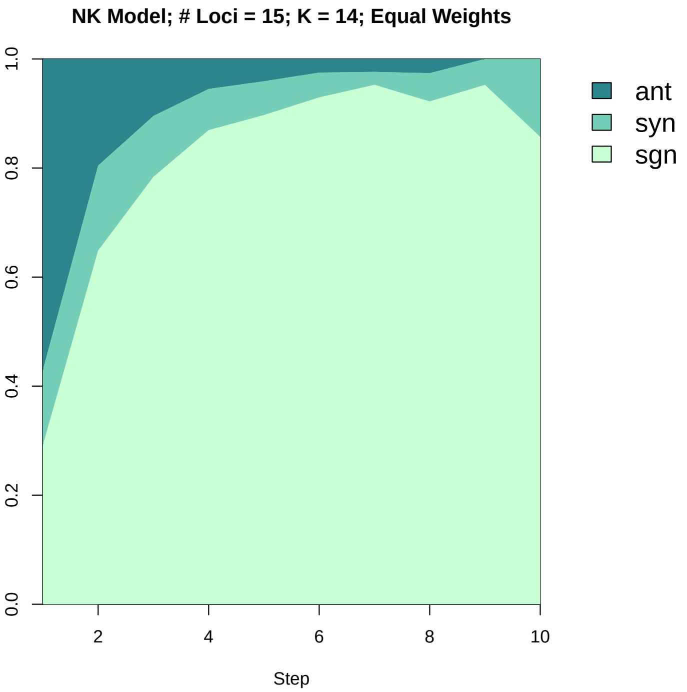
NK Model; # Loci = 15; K = 14; Equal Weights
0.0
0.2
0.4
0.6
0.8
1.0
2
4
6
8
10
Step
ant
syn
sgn

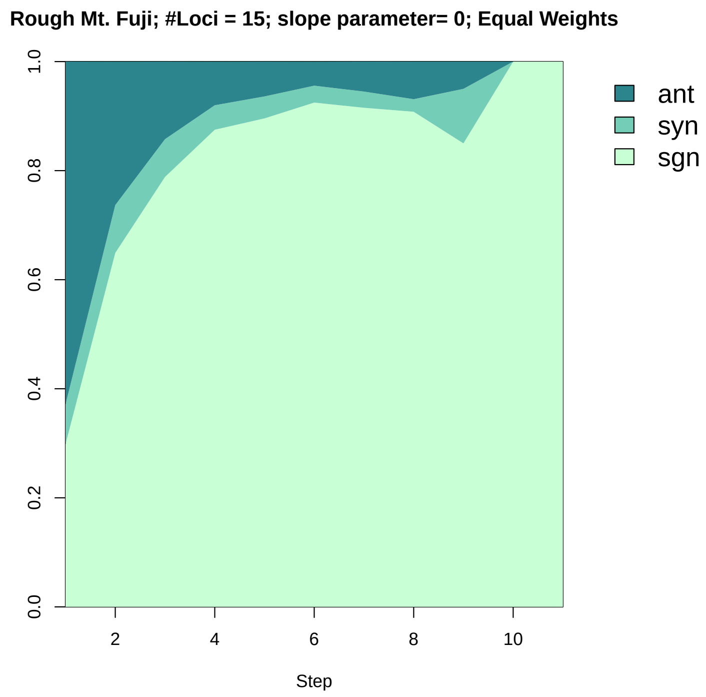
Rough Mt. Fuji; #Loci = 15; slope parameter= 0; Equal Weights
0.0
0.2
0.4
0.6
0.8
1.0
2
4
6
8
10
Step
ant
syn
sgn

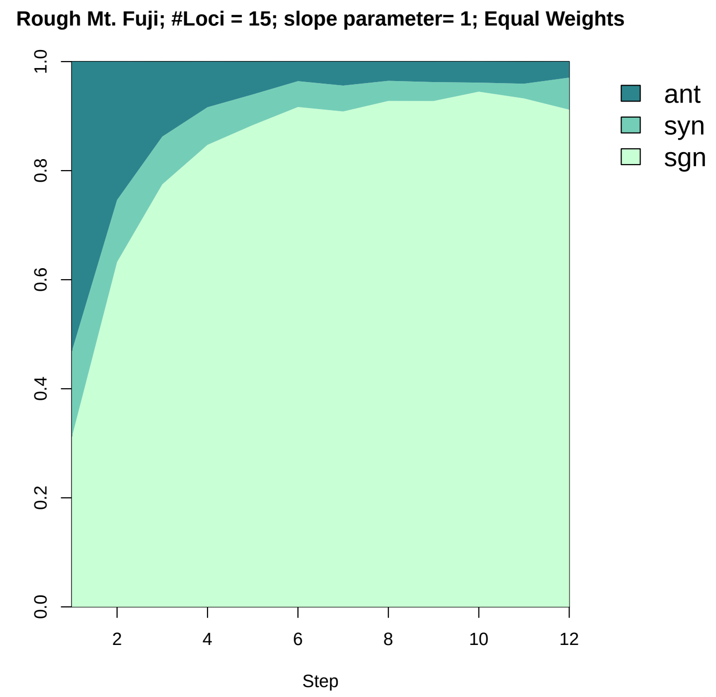
Rough Mt. Fuji; #Loci = 15; slope parameter= 1; Equal Weights
0.0
0.2
0.4
0.6
0.8
1.0
2
4
6
8
10
12
Step
ant
syn
sgn

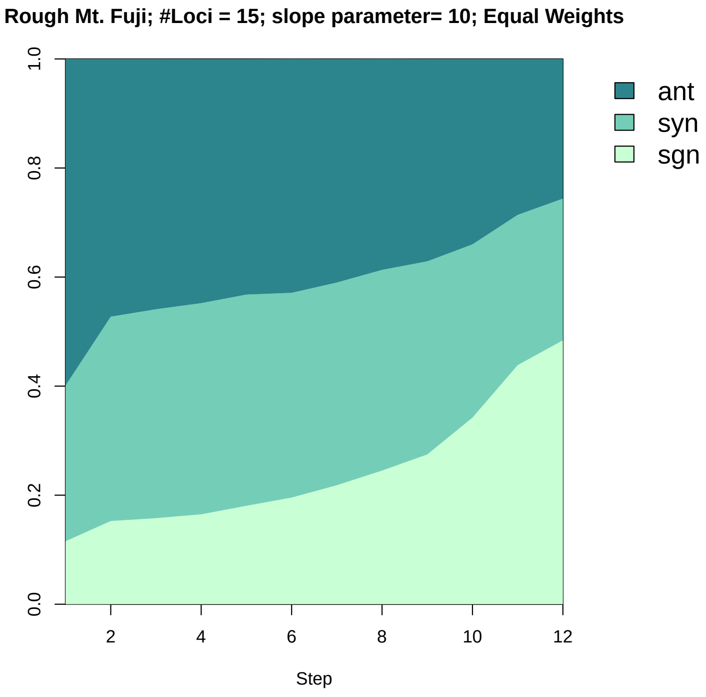
Rough Mt. Fuji; #Loci = 15; slope parameter= 10; Equal Weights
0.0
0.2
0.4
0.6
0.8
1.0
2
4
6
8
10
12
Step
ant
syn
sgn

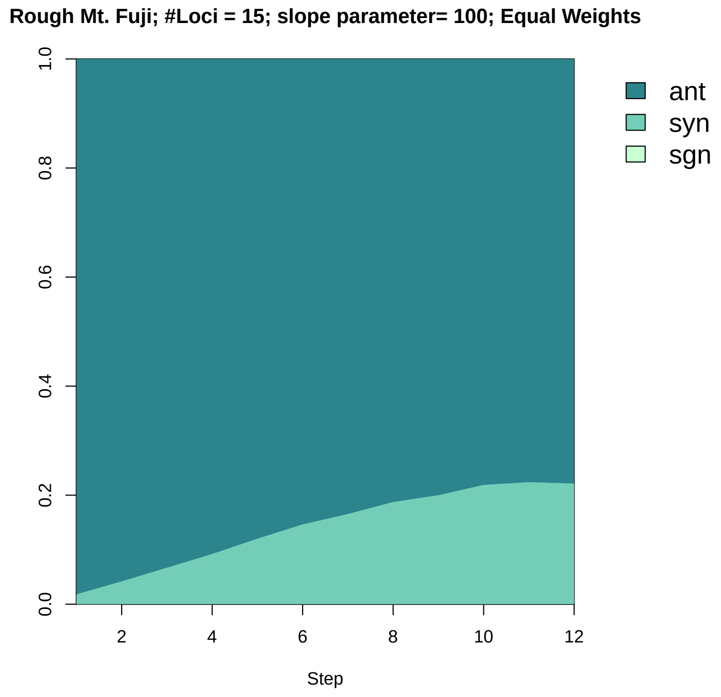
Rough Mt. Fuji; #Loci = 15; slope parameter= 100; Equal Weights
0.0
0.2
0.4
0.6
0.8
1.0
2
4
6
8
10
12
Step
ant
syn
sgn

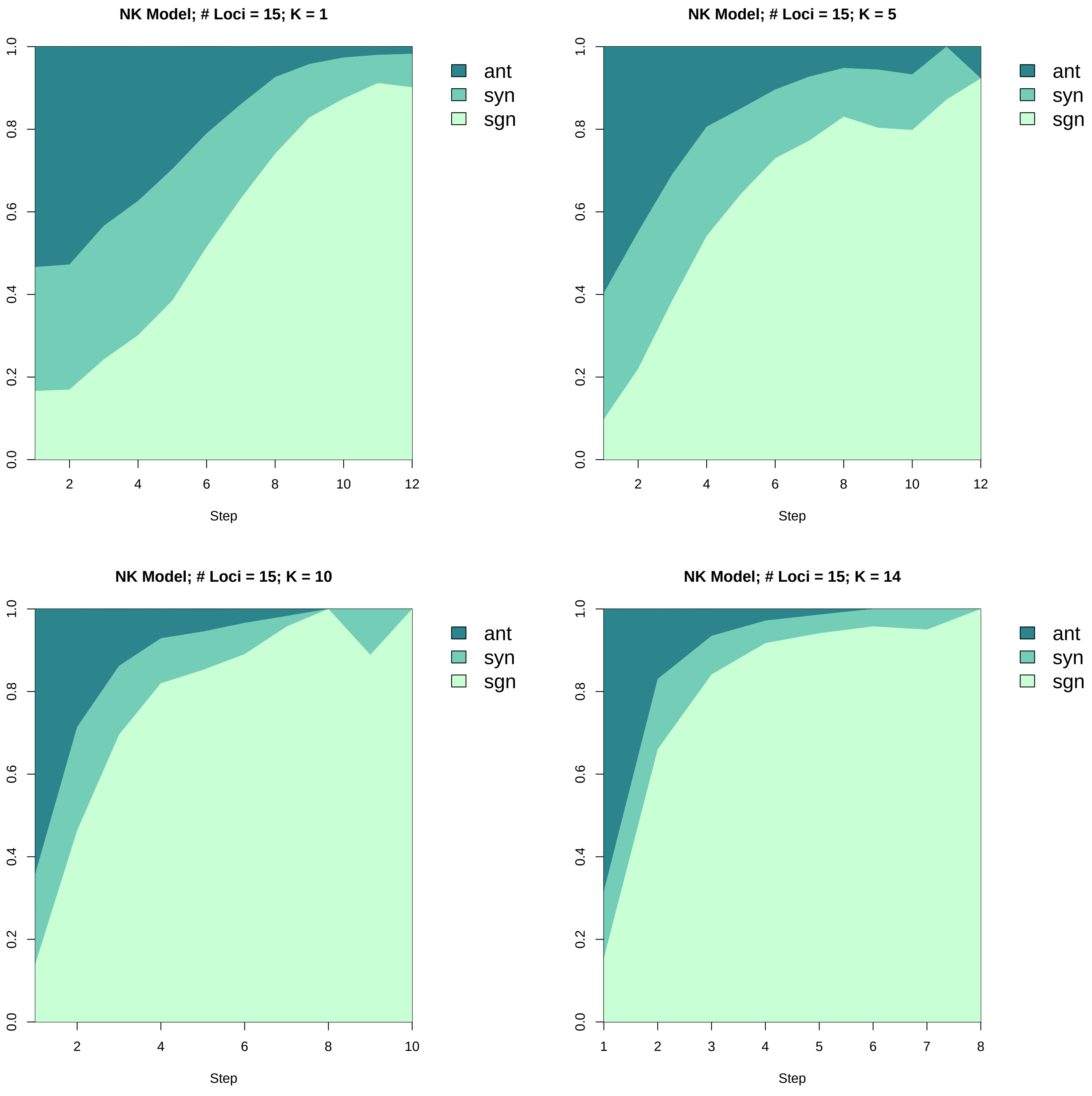
NK Model; # Loci = 15; K = 1
NK Model; # Loci = 15; K = 5
NK Model; # Loci = 15; K = 10
NK Model; # Loci = 15; K = 14
ant
syn
sgn
Step
0.0
0.2
0.4
0.6
0.8
1.0
1
2
3
4
5
6
7
8
10
12

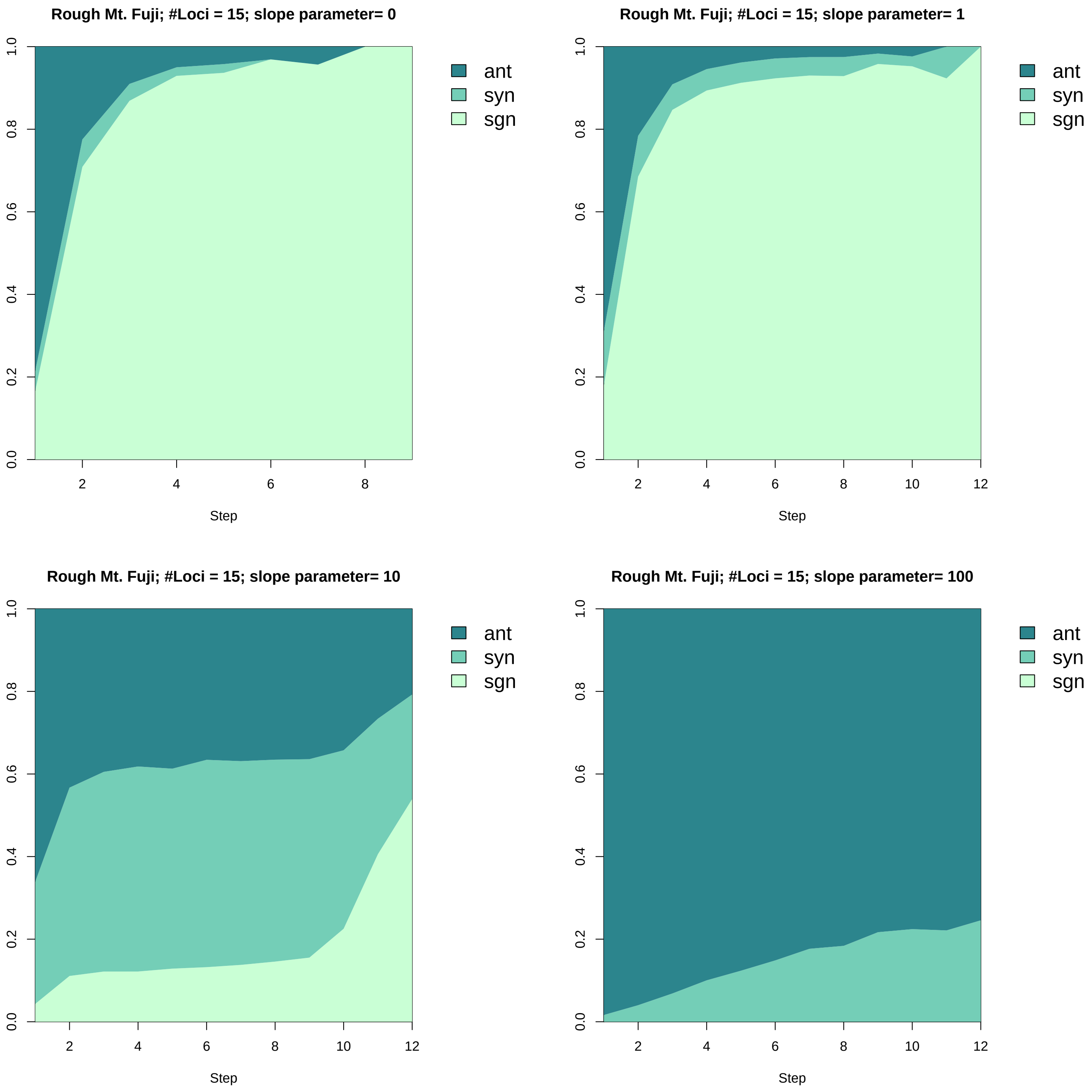
Rough Mt. Fuji; #Loci = 15; slope parameter= 0
Rough Mt. Fuji; #Loci = 15; slope parameter= 1
Rough Mt. Fuji; #Loci = 15; slope parameter= 10
Rough Mt. Fuji; #Loci = 15; slope parameter= 100
ant
syn
sgn
Step
0.0
0.2
0.4
0.6
0.8
1.0
2
4
6
8
10
12
